# In-Situ Preparation of CdTe Quantum Dots Capped with a β-Cyclodextrin-Epichlorohydrin Polymer: Polymer Influence on the Nanocrystal’s Optical Properties

**DOI:** 10.3390/nano8110948

**Published:** 2018-11-17

**Authors:** Rudy Martin-Trasanco, Hilda E. Esparza-Ponce, Pedro D. Ortiz, Diego P. Oyarzun, Cesar Zuñiga, Maria E. Montero-Cabrera, Alain Tundidor-Camba, Guadalupe del C. Pizarro, Ramiro Arratia-Pérez

**Affiliations:** 1 Center for Applyed Nanoscience (CANS), Doctorado de Físicoquímica Molecular Universidad Andres Bello, Av. República 275, Santiago 8370146, Chile; diequim@gmail.com (D.P.O.); cesar.zuniga.c@gmail.com (C.Z.); rarratia@unab.cl (R.A.-P.); 2 Centro de Investigación en Materiales Avanzados S.C, Ave. Miguel de Cervantes 120, Complejo Industrial Chihuahua, 31109 Chihuahua, Mexico; hilda.esparza@cimav.edu.mx (H.E.E.-P.); elena.montero@cimav.edu.mx (M.E.M.-C.); 3 Departamento de Química Inorgánica, Facultad de Química, Pontificia Universidad Catolica de Chile, Avenida Vicuña Mackenna, 4860, Santiago 7820436, Chile; pdortiz@gmail.com; 4 Research Laboratory for Organic Polymers (RLOP), Faculty of Chemistry, Pontifícia Universidad Católica de Chile, Avenida Vicuña Mackenna, Santiago 7820436, Chile; atundido@uc.cl; 5 Department of Chemistry, Technological Metropolitan University, J. P. Alessandri 1242., Santiago 7800003, Chile; pizarroguadalupe048@gmail.com

**Keywords:** CdTe quantum dots, cyclodextrin-epichlorohydrin polymer, surface modification, oswald ripening stage

## Abstract

β-Cyclodextrin (βCD), the less water soluble of the cyclodextrins, has been used as a capping agent in the preparation of semiconductor nanocrystals or quantum dots (QDs). Nevertheless, no reports have been found in the use of the highly water-soluble polymer of this, prepared by the crosslinking of the βCD units with epichlorohydrin in basic medium (βCDP). This polymer, besides to overcome the low solubility of the βCD, increases the inclusion constant of the guest; two parameters that deserve its use as capping agent, instead of the native cyclodextrin. In the present manuscript, we afforded the in-situ aqueous preparation of cadmium telluride (CdTe) QDs capped with βCDP. The polymer influence on the photoluminescent properties of the nanocrystals was analyzed. The βCDP controls the nanocrystals growth during the Oswald ripening stage. Consequently, the CdTe capped βCDP QDs showed lower Stokes-shift values, higher photoluminescent efficiency, and narrower size distribution than for nanocrystals obtained in the absence of polymer. Transmission electron microscopy (TEM) micrographs and energy dispersive X-ray spectroscopy (EDS) analysis revealed the composition and crystallinity of the CdTe QDs. This βCDP capped CdTe QDs is a potential scaffold for the supramolecular modification of QDs surface.

## 1. Introduction 

Quantum dots (QDs) are zero-dimensional semiconductor materials, often prepared by organometallic synthetic routes. This method consists in reflux a metal-organic precursor, i.e., trioctylphosphine (TOP) in a high boiling coordinating solvent, such as trioctylphosphine oxide (TOPO) [1]. Although this method is recognized as the most adequate to prepare high crystalline and monodisperse QDs, the presence of TOPO as a capping agent confers a hydrophobic surface that makes them unavailable to be directly used in aqueous media application. The aqueous compatibility of these QDs is accessed by replacing the TOPO ligand via a ligand exchange methodology, a procedure that attempts against the photoluminescent efficiency [2]. 

The methods for preparing chalcogenide QDs (i.e., ME; M = Zn, Cd or Hg and E = Te, Se, S), in aqueous media, were pioneered by Henglein [3] and Fendler [4] and has been improved by other authors [5,6,7]. The aqueous synthetic approach consists in the injection of a chalcogenide precursor (H_2_E or NaHE) into a refluxing solution of metal-thiol complexes at high pH (8–12), under inert atmosphere (N_2_ or Ar). By extending the time of reflux, the growth of nanocrystals occurs, and QDs with the desired size are obtained [7]. 

The aqueous colloidal synthesis of QDs is an advantageous alternative in comparison to the organometallic ones [8]. Using water as an environmentally friendly solvent, the easily up-scalable procedure, and the ability of the surface functionalization, by using an appropriate capping ligand, are some of the main benefits in the use of the aqueous synthetic route for preparing QDs [2,7,9]. 

The strong and tuneable photoluminescent properties in the visible spectrum (500–750 nm), makes cadmium telluride (CdTe) QDs a classic II-VI QDs type, which are often prepared in water [10]. Due to the nanometric size, QDs tend to aggregate or decompose, and therefore, their surface should be protected and passivated with a capping or by using stabilizing ligands. In fact, this is the role of mercapto acids used during CdTe QDs preparation, which, additionally, to avoid the aggregation by electrostatic repulsion, passivates the Cd^2+^ on the QDs surface by forming a stable -S-Cd bond [2]. 

The use of polymers as capping agents improves the stability of QDs and provides their chemical functionalities without affecting the QDs’ photoluminescent properties [7]. Three methodologies can be afforded for the aqueous preparation of QDs capped with polymers: (i) attaching the polymer to the previously prepared nanocrystals by direct mixing of them “grafting-to”, (ii) capping the QDs with monomer which will be further polymerized “grafting-from” and (iii) preparing QDs in the presence of polymer in-situ. From the synthetic point of view, the latter methodology is the friendliest, since the extensive purification steps required in the first two are avoided [11]. 

Cyclodextrins (CDs) family compounds are enzymatically produced from starch and are composed of six, seven, or eight glucopyranoside units (α, β, and γCD, respectively) linked by an α-(1→4) linkage [12,13]. The arrangement of the glucopyranose units leads to a cyclic oligosaccharide with a hydrophobic central cavity. This unique structure allows them to form stable inclusion complexes with a wide variety of guests containing hydrophobic moieties [14,15]. Therefore, cyclodextrins capped QDs are excellent candidate systems to prepare selective and sensitive nanodevices for various targets, since it combines the molecular recognition features of CDs and the fluorescence properties of QDs [16]. Sensors based on natural and derivative cyclodextrins capped QDs have been prepared for biomedical and environmental applications [16,17,18,19]. 

Among the three CDs, βCD is the most commercially available and its cavity is of adequate size to form stable inclusion complexes with a wide variety of drugs. However, it has the drawback of scarce water solubility (1.85 g/100 mL, at 25 °C) [20]. The low solubility of βCD can be overcome by its crosslinking with epichlorohydrin (βCDP) in an alkaline medium [21,22]. The resulted polymer not only increases the solubility of the guest, but also increases the inclusion constant with respect to the native βCD [14]. 

Recently, we prepared in-situ gold nanoparticles capped with βCDP [21,23]. In the herein presented manuscript, we went one step further and prepared CdTe QDs capped with βCDP, in aqueous medium. Additionally, the effect of the βCDP on the photoluminescent properties of QDs was analyzed. These βCDP capped CdTe QDs represent a breakthrough in the design of new scaffolds for preparing functionalized fluorescent nanodevices based on supramolecular interactions. 

## 2. Experimental Part 

### 2.1. Chemicals

Cadmium chloride (99%), mercaptopropionic acid (97%), tellurium (powder, 99%), sodium borohydride (98%), epichlorohydrin, epi, (98%) and β-cyclodextrin hydrated, βCD (97%) were purchased from Sigma-Aldrich (Sigma-Aldrich Química Ltda., Santiago de Chile, Chile) and were used without further purification. Fluorescein and ethanol, both of spectroscopic grade, were also purchased from Sigma-Aldrich (Sigma-Aldrich Química Ltda., Santiago de Chile, Chile). The β-cyclodextrin-epichlorohydrin polymer (βCDP, Mw ≈ 12.5 kDa, determined by static light scattering and 3.5 nm of hydrodynamic diameter) was prepared as previously reported [14]. Freshly purified MilliQ water (18 mΩ/cm) was employed in all the synthesis and procedures. *Aqua regia* (3:1 hydrochloric acid/nitric acid, *v*/*v*) was used to clean all the glassware before its use (Caution: *aqua regia* is toxic and corrosive and must be handled with care).

### 2.2. Preparation of Telluride Precursor (NaHTe) and β-Cyclodextrin Polymer Capped Cadmium Telluride Quantum Dots (CdTe@MPA@βCDP)

Telluride precursor (NaHTe) was prepared by reducing tellurium with sodium borohydride under argon atmosphere, as reported elsewhere [24]. Aqueous colloidal CdTe QDs were prepared in the absence and presence of the βCDP according to the in-situ methodology. In a three necks round bottom flask equipped with a condenser, cadmium chloride (430 mg, 2.34 mmol) was dissolved, under stirring, in a water solution (120 mL) of βCDP (120 mg). Mercaptopropionic acid (266 μL, 3.04 mmol) was added to the solution and the pH was adjusted to 11.0 with NaOH (0.1 mol/L). The resulted solution was exhaustively deoxygenated with argon (30 min) and a freshly prepared NaHTe (1760 μL, 1.16 mmol) was added under stirring and in a gentle argon flow. The reaction mixture was set to reflux and the evolution of the QDs formation was monitored by recording the UV-Vis and fluorescent spectra of samples collected at certain reaction times. Each collected sample was set to dialyze in a Spectrapore^®^ dialysis membrane (cutoff 14 kDa) in order to remove the excess of polymer and chemicals resulting from the reaction. For comparison purposes, CdTe QDs were also prepared under the same conditions but in the absence of the βCDP. 

### 2.3. Spectroscopy 

UV-Vis Spectrophotometry: The UV-Vis spectra were recorded on a Perkin-Elmer Lambda-35 spectrophotometer (Perkin-Elmer, Waltham, MA, US) with a resolution of 1 nm employing quartz cuvettes with 1 cm of path length. 

Fluorescent spectrophotometry: The fluorescent spectra were recorded on a Shimadzu RF-5301PC spectrofluorophotometer (Shimadzu, Kioto, Japan) with a resolution of 1 nm using Hëllma Analytics quartz cuvettes (Hellma USA, NY, US) of 1 cm path length. 

ATR-FT-IR Spectroscopy: The FT-IR spectrums were recorded on a Perkin-Elmer Spectrum-Two spectrometer (Perkin-Elmer, Waltham, MA, US) with a UATR unit coupled. The sample was directly positioned over the diamond, pressed until 30% of the total supported pressure and scanned in the range of 4000 to 500 cm^−1^ with a resolution of 1 cm^−1^.

### 2.4. Quantum Yield Measurements 

The photoluminescent properties of the as-prepared QDs were determined according to the consideration reported by Eychmüller and Resch-Genger [25]. Fluorescein-F27 was chosen as a reference ($\phi_{F}$ = 92%) [26]. The absorption and emission spectra of six fluorescein solutions and six solutions of the selected as-prepared CdTe QDs were recorded and the quantum yield of the latter was calculated according to Equation (1)):

(1) $$\phi_{QDs}=\phi_{F}\frac{m_{QDs}}{m_{F}}\cdot \frac{n_{QDs}^{2}}{n_{F}^{2}}$$

where QDs and F subscripts indicates quantum dots and fluorescein-27, respectively, ϕ is the corresponding quantum yield, and m is the slope of the straight line resulting from plotting the area of the fluorescence spectrum (corrected from the solvent emission) versus the absorbance (<0.1 a.u) at the excitation wavelength (465 nm) for each the six prepared samples of QDs and fluorescein. 

### 2.5. Transmission Electron Microscopy (TEM)

The transmission electron microscopy (TEM) micrograph of the as-prepared QDs was taken in a JEOL transmission electron microscope, model JEM 2200FS (JEOL LTD, Akishima, Tokio, Japan), equipped with a CCD camera MSC 2048X2048 GATAN and EDS for different magnifications. The images were obtained by using dark and bright field TEM and high angle annular dark field scanning TEM (HAADF-STEM) as imaging modes. The samples were placed on 400 mesh carbon-coated copper grids. The TEM micrographs were analyzed using the software ImageJ V. 1.46r [27]. 

## 3. Results and Discussion

### 3.1. Synthesis and Optical Properties of βCDP Capped QDs

**Scheme 1:**
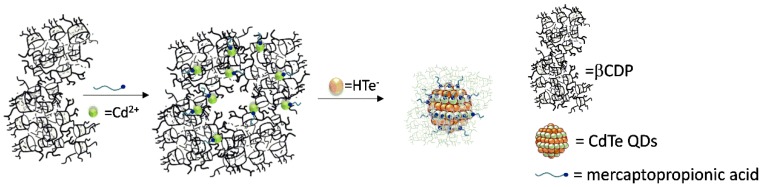
Preparation of cadmium telluride (CdTe) quantum dots (QDs) in-situ of βCDP solution. Mercaptopropionic acid coordinates Cd^2+^in the presence of β-Cyclodextrin epichlorohydrin (βCDP). Telluride precursor (NaHTe) is added to the solution and submitted to reflux to start the formation and growth of the CdTe nanocrystals.

In order to prepare a supramolecular fluorescent scaffold and take advantage of the ability of βCD to form inclusion complexes with a wide variety of guest, the aqueous synthesis of the CdTe QDs were carried out in the presence of the βCDP. The βCDP, additionally, served as a supramolecular host on the QDs surface, and acted as a capping agent. The βCDP capped nanocrystals (CdTe@MPA@βCDP) were prepared by the in-situ methodology according to Scheme 1.

**Figure 1:**
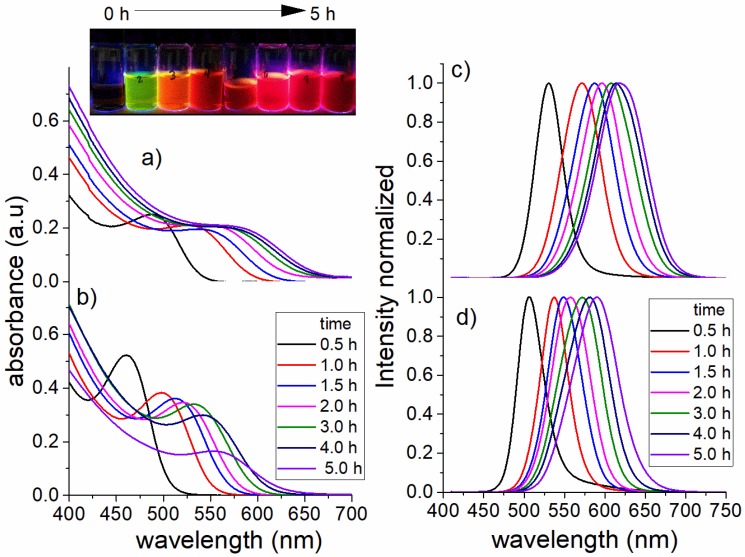
UV-Vis (**a**,**b**) and normalized emission spectra (**c**,**d**) of CdTe@mercaptopropionic acid (MPA) (**a**–**c**)) and CdTe@MPA@βCDP (**b**–**d**). Inset: picture of samples of CdTe@MPA@βCDP reaction mixture collected at different reaction time, irradiated with UV-light at 365 nm.

The addition of mercaptopropionic acid (MPA), to the Cd^2+^ solutions provoked the appearance of turbidity. This turbidity corresponds to the neutral and poorly water-soluble complex of Cd-MPA [28]. After the addition of NaHTe, the solutions became brownish and rapidly changed to a clear yellow. The reaction mixture changed the color during the first two hours of reaction (Figure 1, picture inset) and afterward kept the final orange color. These changes in color were the first indicative of CdTe QDs formation. 

#### 3.1.1. Effects on the First Exciton and Fluorescence Spectra

The formation of QDs was verified by recording the UV-Vis and fluorescence spectra to both reaction mixtures at certain time intervals (0.5–5 h). Figure 1 shows the UV-Vis and normalized emission spectra of the CdTe@MPA (Figure 1a,c) and CdTe@MPA@βCDP QDs (Figure 1b,d). In both cases, the UV-Vis spectra show the excitonic absorption band characteristic of semiconductor nanocrystals. In the absence of βCDP, this band was shifted from 488 to 590 nm with the ongoing reaction, while in the presence of the polymer it was always recorded at shorter wavelengths for the same time intervals (from 460 nm to 577 nm). 

The fluorescent spectra of both reaction samples showed maximum emission bands between 530 and 617 nm for CdTe@MPA and within 506 nm and 575 nm for CdTe@MPA@βCDP. In both cases, the UV-Vis and photoluminescent spectra show the typical behavior of CdTe nanocrystals growth with the time of reflux. With the growth of the CdTe QDs, the band-gap of the semiconductor decreased, and therefore, the first excitonic and the emission bands are red shifted. Interestingly, for the same time intervals, both the excitonic and the emission bands of CdTe@MPA@βCDP were recorded at lower wavelengths, suggesting a smaller size of these at the same reaction time. 

In CdTe, colloidal quantum dots, the dependence of the band-gap with the nanocrystal size have been successfully addressed from the theoretical approach [29,30]. In our case, the diameter (D, nm) of the as-prepared CdTe QDs was determined from the wavelength of the first excitonic absorption peak (λ) of the different aliquots, accordingly to the empirical equation proposed by Peng et al. (Equation (2)) [31]:

(2) $$D_{QDs}^{CdTe}=\left(9.8127\times 10^{-7}\right)\lambda^{3}\;-\;\left(1.7147\times 10^{-3}\right)\lambda^{2}\;+\;\left(1.0064\right)\lambda \;-\;\left(194.84\right)$$

**Figure 2:**
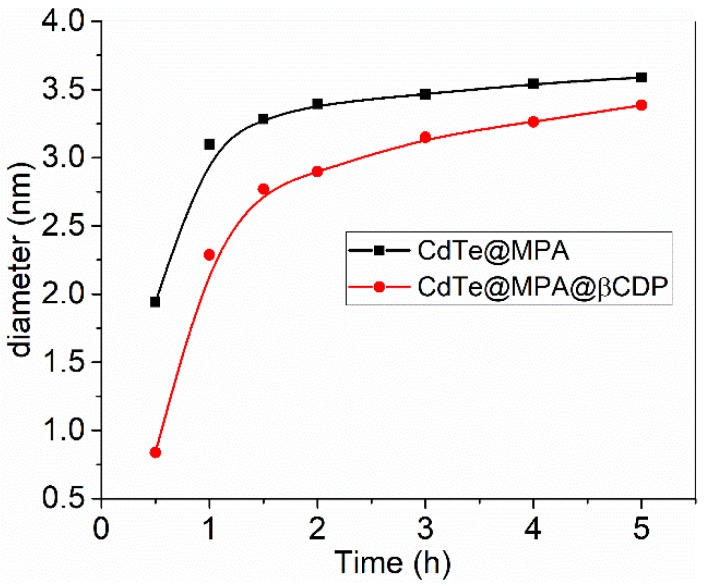
Size-dependence of CdTe QDs prepared in the absence and presence of βCDP.

Figure 2 shows the size-dependence of CdTe QDs prepared in the absence and presence of βCDP, with the reaction time. Two main stages in nanocrystal growth can be observed: (i) fast growth, at shorter reaction times within the first 60 min and 90 min for CdTe@MPA and CdTe@MPA@βCDP, respectively, and (ii) a slower growth rate at longer times, which reach a plateau for CdTe@MPA, while for CdTe@MPA@βCDP remains growing at a lower rate. A marked difference in size between QDs prepared in the presence and absence of the polymer is observed at shorter reaction times (first 2 h). This result suggested that the βCDP has a direct influence on the growing mechanism of CdTe QDs.

#### 3.1.2. Stokes-Shift and Full Width at Half Maximum (FWHM) of the Emission Band

**Figure 3:**
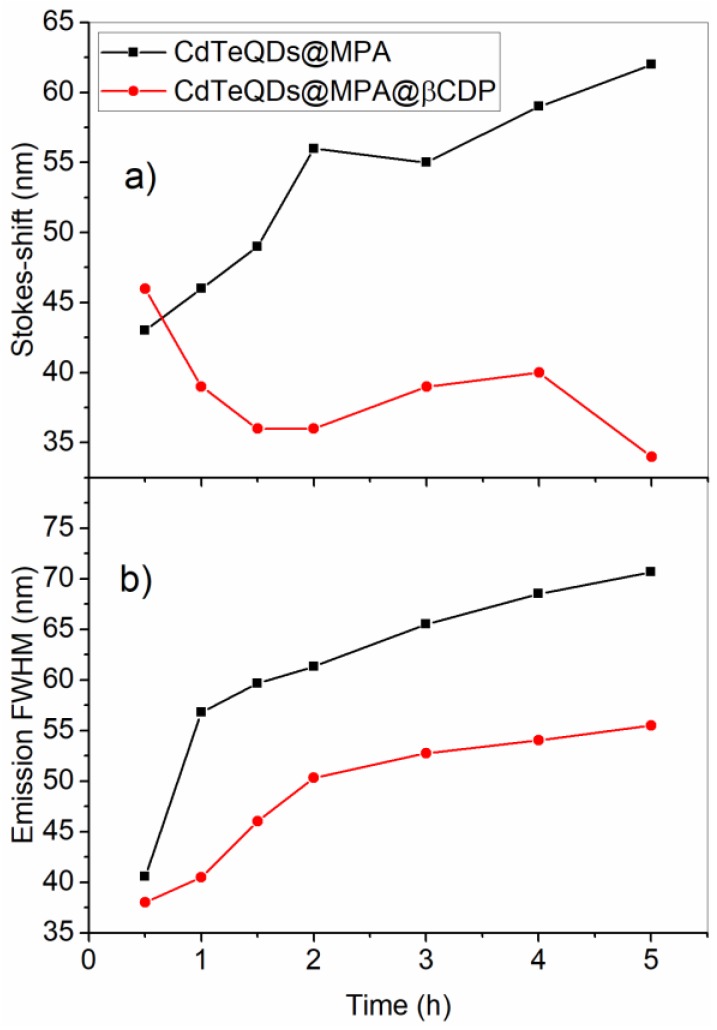
Graphs of Stokes-shift (**a**) and Full Width at Half Maximum (FWHM) (**b**) at different reaction time in the absence (squares) and presence (circles) of βCDP.

The Stokes-shift and the Full Width at Half of the Maximum (FWHM) intensity of the emission band are two important parameters that characterize nanocrystals [28]. Stokes-shift is mainly related to the distribution of defects (traps-states) in the nanocrystal structure and at its surface (shallow surface traps) [28,32,33]: the larger the number of defects, the larger the Stokes-shift. FWHM is directly proportional to the polydispersity in the size of the nanocrystals. Figure 3 shows the trend in the Stokes-shift and the FWHM values determined to CdTe@MPA and CdTe@MPA@βCDP.

In the absence of βCDP, the Stokes-shift values increased during the ongoing reaction. Contrary to this, in the presence of βCDP, this parameter decreased within the first 90 min. Although the FWHM values increased in both cases, in the presence of βCDP it was less marked, indicating a narrower size distribution. According to these results, it is important to stress that: QDs prepared in the presence of βCDP were smaller within the initial stage of the reaction, had an apparently higher quality, and had a less size polydispersity than those prepared in the absence of the polymer.

The highest emission intensities were achieved after 60 and 90 min of reflux with photoluminescent efficiencies ($\phi_{QDs}$) of 13% and 18% for CdTe@MPA (λ^emis^ = 571 nm) and CdTe@MPA@βCDP (λ^emiss^ = 549 nm), respectively (Figure S1 in Supplementary Data). These values are in agreement with others reported for CdTe QDs prepared by the aqueous approach [7]. The higher $\phi_{QDs}$ of CdTe@MPA@βCDP confirms the better quality of these with regards to CdTe@MPA, as was suggested by the lower Stokes-shift and FWHM of the emission values of the former. For these samples, CdTe@MPA, at 60 min, and CdTe@MPA@βCDP, at 90 min, the as-prepared QDs were characterized by ATR-FTIR and Transmission Electron Microscopy (TEM). 

### 3.2. ATR-FTIR Characterization of the In-Situ Prepared CdTe@MPA QDs Capped with βCDP 

**Figure 4:**
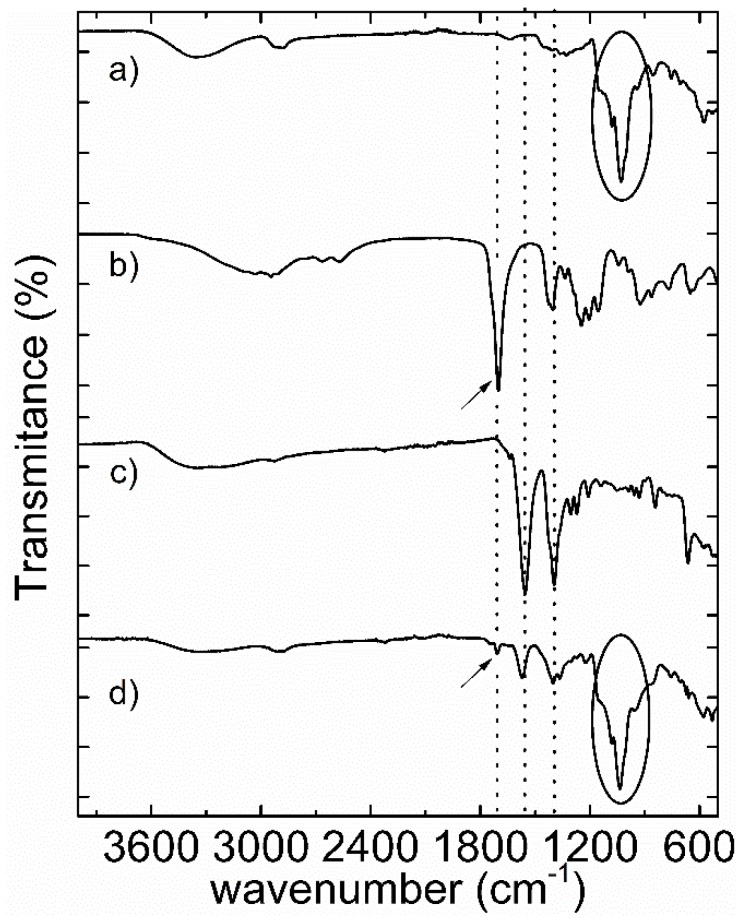
ATR-FTIR of βCDP (**a**) MPA (**b**) CdTe@MPA (**c**) and CdTe@MPA@βCDP (**d**).

Figure 4 shows the ATR-FTIR spectra of the highest emission fraction of CdTe@MPA and CdTe@MPA@βCDP, isolated at 60 min and 90 min after starting the reaction, respectively. The ATR-FTIR of βCDP and MPA were also recorded for comparison purposes. 

The spectrum recorded to the βCDP (Figure 4a) showed a strong and wide signal at 1037 cm^−1^. This signal was assigned to the superposition of the stretching and bending vibration modes of the α-glucopiranoside rings of the βCD in βCDP [34]. In the spectrum of CdTe@MPA@βCDP (Figure 4d) this signal was also recorded. The broad band recorded at 3350 cm^−1^ corresponded to the stretching of the hydroxyl groups (−OH) in the βCDP. The spectrum of MPA (Figure 4b) shows a sharp and intense band at 1706 cm^−1^ which corresponds to υ(CO) of carboxylic acid. In this spectrum, additional to the carbonyl band, a small band was recorded at 2569 cm^−1^. This band is characteristic of υ(−SH) of the thiol group in the MPA. 

In the FT-IR spectra of CdTe@MPA (Figure 4c) and CdTe@MPA@βCDP (Figure 4d) the υ(−SH) band was not recorded. The absence of this band indicates that the sulphur in MPA was coordinating the Cd^2+^, at the QDs surface, as thiolate. Additionally, two bands at 1557 and 1396 cm^−1^, were recorded in both spectra, These bands correspond to $\upsilon^{as}\left(OCO\right)$ and $\upsilon^{s}\left(OCO\right)$, respectively, of the carboxylate anion of MPA. The presence of the carboxylate anion was due to the basic medium required for the preparation of CdTe QDs.

**Scheme 2:**
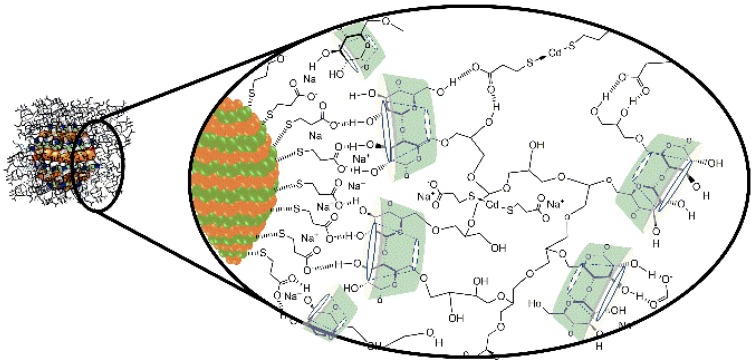
Representation of proposed cooperative hydrogen bond interaction between MPA at CdTe surface and the βCDP and the hindered access of Cd^2+^-MPA to the QDs surface, by the βCDP.

In the FT-IR spectrum of CdTe@MPA@βCDP (Figure 4d), additional to the $\upsilon^{as}\left(OCO\right)$ and $\upsilon^{s}\left(OCO\right)$ bands, a third, less intense band was recorded at 1709 cm^−1^. As can be noted, this band is near the position of the υ(CO) of carboxylic acid in MPA. The presence of this carbonyl stretching band in the CdTe@MPA@βCDP FT-IR spectrum, which can only arise from the protonation of the carboxylate anion, suggests that the polymer is interacting with the carboxylate anion at the QDs surface, most likely through a cooperatively hydrogen bond. This hydrogen bond interaction between the carboxylate anion in MPA and the –OH in the βCDP is represented in Scheme 2 (see below). 

### 3.3. High Resolution Transmission Electron Microscopy Characterization (HRTEM)

**Figure 5:**
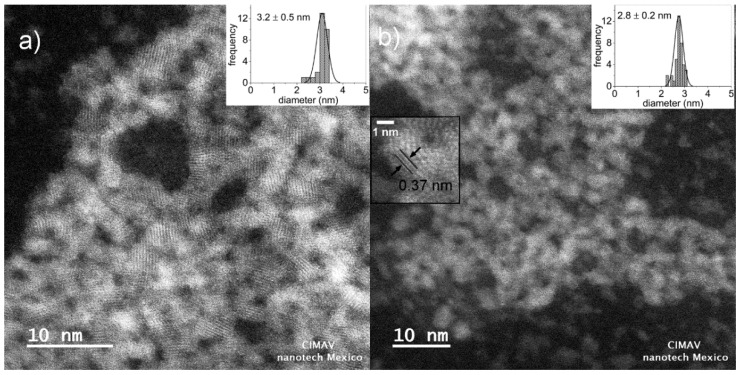
Transmission electron microscopy (TEM) micrograph of CdTe@MPA (**a**) and CdTe@MPA@βCDP (**b**) and their respective histograms (inset).

HRTEM was used to characterize the size and morphology of the as-prepared CdTe QDs with the higher emission intensity (60 and 90 min upon starting the reaction for CdTe@MPA and CdTe@MPA@βCDP, respectively). In both micrographs (Figure 5), aggregates of CdTe nanocrystals are observed. It is well known that thiol-capped nanocrystals prepared by the aqueous approach tends to aggregate upon deposited on the TEM grids [28,35]. This situation is clearly observed in the QDs prepared in the absence of βCDP (Figure 5a). 

The as-prepared QDs obtained in the presence of βCDP, although with certain aggregates, has better-defined boundaries (Figure 5b) than those prepared in the absence of this (Figure 5a), most likely due to the presence of the polymer protecting the surface. The size determined from the micrograph (histograms inset) was estimated by measuring the diameter of QDs with resolved boundaries. QDs prepared, at 60 min, in the absence of βCDP had a diameter of 3.2 ± 0.5 nm, whereas those prepared, at 90 min, in the presence of βCDP showed diameters of 2.8 ± 0.2 nm. This is in agreement with the results obtained from the empirical equation proposed by Peng et al. [31] at these time of reaction (90 min for CdTe@MPA@βCDP and 60 min for CdTe@MPA). 

Interestingly, in both TEM micrographs, a well-defined lattice plane was observed. The average in plane distances (Figure 5, image inset) was 0.37 nm. This value is in agreement with the plane distance of the cubic [111] facets of CdTe [36] and confirm, together with the Energy Dispersive X-ray Spectroscopy analysis (Figure S2 in Supplementary Data), the composition and crystallinity of the prepared CdTe@βCDP QDs. From the above mentioned results, it can be observed that, in the presence of βCDP, the growth of QDs occurs at a lower rate.

The mechanism proposed for CdTe nanocrystal’s growth in aqueous media consists of a first stage involving the fast formation of the nucleus and a second stage corresponding to the Oswald ripening, which proceeds at slower rates [7]. It can be stated that, during the growth stage, the rate of nanocrystals growth depends on the electrostatic nature of the ligand adsorbed and the diffuse layer through which the precursors (Cd-MPA and HTe^-^) should diffuse to enable the nanocrystal surface to grow [37].

Accordingly to this, it can be stated that:

(i) The presence of the βCDP on the CdTe QDs surface brings in an additional diffusion step to the precursors (Cd-MPA and HTe^-^) within the polymer network before reaching the QDs surface.
(ii) The presence of the βCDP attached to MPA, on the QDs surface, alters the electrostatic nature of the QDs surface. These situations are described in Scheme 2.

Both features should decrease the growth rate of nanocrystals during the Oswald ripening stage provoking the aforementioned results in size and quality of CdTe QDs. Nevertheless, detailed studies will be necessary to elucidate how the βCDP exert this role. 

## 4. Conclusions

Cadmium telluride nanocrystals capped with a βCDP polymer were obtained through the aqueous synthesis approach. For the initial time intervals of reaction, QDs prepared in the presence of the polymer were smaller than prepared in the absence of this. The Stokes-shift of nanocrystals prepared in the presence of βCDP decreased by 10 nm during the ongoing reaction, while in the absence of this increased by 20 nm. ATR-FTIR results suggested that the βCDP polymer interacts with QDs surface through a cooperative hydrogen bond interaction between MPA, and the hydroxyl groups in βCDP. The higher photoluminescent efficiency of QDs was achieved at 60 and 90 min. when CdTe@MPA and CdTe@MPA@βCDP started to reflux, respectively. The presence of βCDP controls the nanocrystals growth during the Oswald ripening stage and QDs with smaller Stokes-shift values, higher photoluminescent efficiency, and narrower size distribution, with respect to those prepared in the absence of this, were obtained.

## Supplementary Materials

The following are available online at http://www.mdpi.com/2079-4991/8/11/948/s1, Figure S1: Fluorescent spectra of samples taken at different time of reflux for CdTe QDs prepared in the absence (up) and presence (down) of βCDP. Figure S2: EDS analysis of CdTe@MPA@βCDP sample, collected at 90 min. of reflux.
