# Supplementary material for: In-Situ Preparation of CdTe Quantum Dots Capped with a β-Cyclodextrin-Epichlorohydrin Polymer: Polymer Influence on the Nanocrystal’s Optical Properties
Source: Nanomaterials (Basel). 2018 Nov 17;8(11):948. doi: 10.3390/nano8110948 (PMC6267504; doi:10.3390/nano8110948)
Supplement: Supplementary file 1 [file nanomaterials-08-00948-s001.pdf]

In-situ Preparation of CdTe Quantum Dots Capped with a-cyclodextrin-Epichlorohydrin Polymer: Polymer Influence on the Nanocrystal's Optical Properties

Rudy Martin-Trasanco[a], Hilda E. Esparza-Ponce[b], Pedro D. Ortiz[c], Diego Oyarzun[a], Cesar Zuñiga[a], Maria E. Montero-Cabrera[b], Alain Tundidor-Camba[d], Guadalupe del C. Pizarro[e], Ramiro Arratia-Pérez[a]

[a.] *Center for applyed Nanoscience (CANS), Doctorado de Físicoquímica Molecular Universidad Andres Bello, Av. República 275, Santiago 8370146, Chile. Telf. +56 2 661 8253 E-mail: ruquim@gmail.com; Tel: +56 2 2661 8253.*
[b.] *Centro de Investigación en Materiales Avanzados S.C, Ave. Miguel de Cervantes 120, Complejo Industrial Chihuahua, Chihuahua, Chihuahua, México. C.P:31109*
[c.] *Departamento de Química Inorgánica, Facultad de Química, Pontificia Universidad Catolica de Chile, Avenida Vicuña Mackenna, 4860, Santiago 7820436, ChileUniversidad Tecnológica Metropolitana, Santiago, Chile*
[d.] *Research Laboratory for Organic Polymers (RLOP), Faculty of Chemistry, Pontifícia Universidad Católica de Chile, Avenida Vicuña Mackenna, Santiago 7820436, Chile*
[e.] *Department of Chemistry, Technological Metropolitan University, J. P. Alessandri 1242. Santiago, Chile*

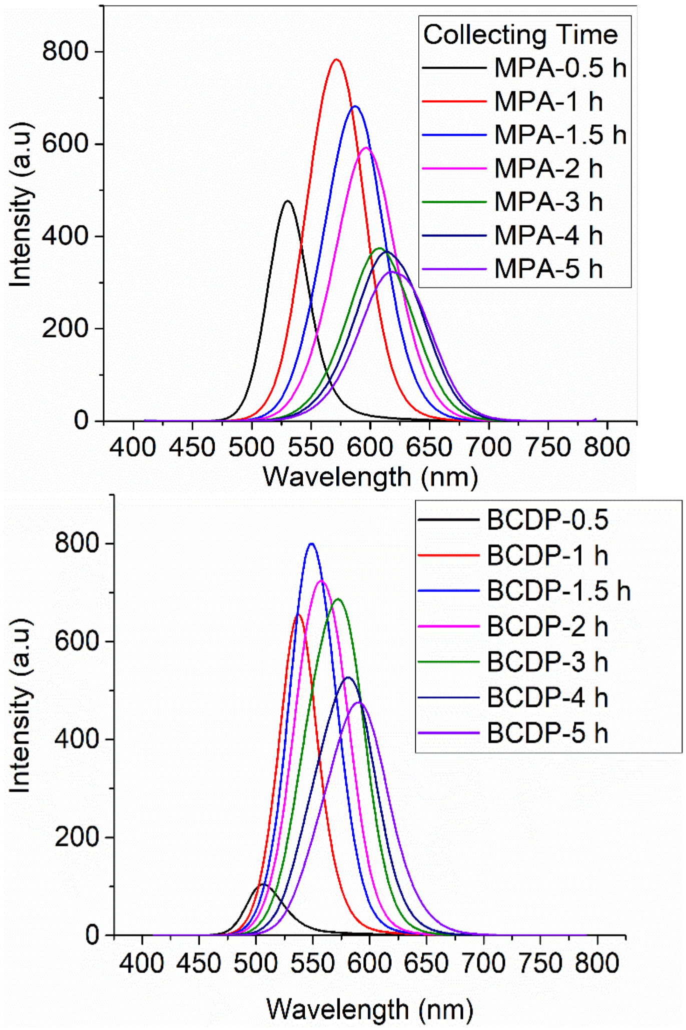

Figure. S1. Fluorescent spectra of samples taken to the reaction at different time of reflux cadmium telluride (CdTe) quantum dots (QDs) prepared in absence (up) and presence (down) of βCDP.

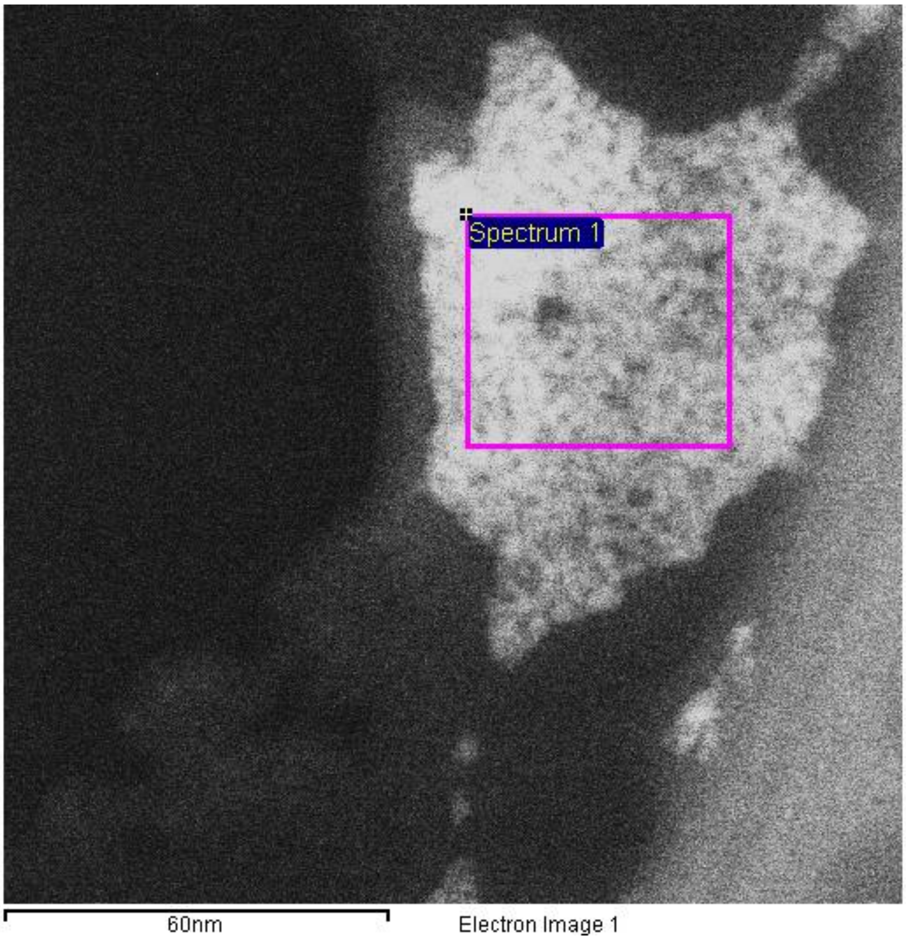

Processing option : All elements analyzed (Normalized). All results are atomic %.

Spectrum	In stats.	C	S	Cd	Te
Spectrum 1	Yes	97.79	0.54	1.22	0.45
Mean		97.79	0.54	1.22	0.45
Std. deviation		0.00	0.00	0.00	0.00
Max.		97.79	0.54	1.22	0.45
Min.		97.79	0.54	1.22	0.45

Figure. S2. EDS analysis of CdTe@ mercaptopropionic acid (MPA)@ β-Cyclodextrin-epichlorohydrin (βCDP) sample, collected at 90 min. of reflux.
